# What the *Fika*? Implementation of Swedish Coffee Breaks During Emergency Medicine Conference

**DOI:** 10.5811/westjem.18462

**Published:** 2024-06-20

**Authors:** Jesse Zane Kellar, Hanna Barrett, Jaclyn Floyd, Michelle Kim, Matthias Barden, Jason An, Ashley Garispe, Matthew Hysell

**Affiliations:** * Saint Agnes Medical Center, Department of Emergency Medicine, Fresno, California; † Riverside Community Hospital, Department of Emergency Medicine, Riverside, California; ‡ Eisenhower Health, Emergency Medicine Residency, Rancho Mirage, California; § Emergency Medicine Residency Corewell Health South, Graduate Medical Education Department, St. Joseph, Michigan

## Abstract

**Introduction:**

In this study we aimed to investigate the effects of incorporating Swedish-style *fika* (coffee) breaks into the didactic schedule of emergency medicine residents on their sleepiness levels during didactic sessions. Fika is a Swedish tradition that involves a deliberate decision to take a break during the workday and usually involves pastries and coffee. We used the Karolinska Sleepiness Scale to assess changes in sleepiness levels before and after the implementation of *fika* breaks.

**Methods:**

The study design involved a randomized crossover trial approach, with data collected from emergency medicine residents over a specific period. This approach was done to minimize confounding and to be statistically efficient.

**Results:**

Results revealed the average sleepiness scale was 4.6 and 5.5 on *fika* and control days, respectively (*P* = 0.004).

**Conclusion:**

Integration of *fika* breaks positively influenced sleepiness levels, thus potentially enhancing the educational experience during residency didactics.

Population Health Research Capsule What do we already know about this issue?

*While interactive sessions and flipped classrooms have been implemented to optimize learning during residency conferences, little is known about how to optimize breaks.*

What was the research question?

*Does implementation of a Swedish fika break improve the level of alertness for emergency medicine residents during conference?*

What was the major finding of the study?

*Average sleepiness on the Karolinska Sleepiness Scale improved from 5.5 on control days to 4.6 on fika days (p = 0.004).*

How does this improve population health?

*This study highlights the importance of structured conference breaks leading to more alert residents and hopefully a higher quality learning environment.*

## INTRODUCTION

Emergency medicine (EM) residency is known for its demanding schedules and high-stress environment. The intensity of residency training can lead to stress, fatigue, and reduced well-being.^1^ At the same time, didactics play a crucial role in providing residents with the necessary knowledge and skills to deliver high-quality patient care and are a key component of learning advancement. Weekly conference sessions may vary in length from program to program but typically comprise five hours of protected time devoted to learning fundamental EM content every week. Much work has been done in recent years to improve the quality of these conferences, such as implementing shorter lectures, interactive sessions, team-based learning, and flipped classrooms.^2^ However, little work has focused on mitigating resident fatigue and decreased attention at the end of the conference session. Research in adult learning data reveals that the attention span of the adult learner decreases dramatically after 15–20 minutes.^3^ Consequently, it is not difficult to assume that after three or four hours of conference, the attention span of the average adult learner has been spent. One possible way to address these challenges is to incorporate breaks in conference days inspired by the Swedish custom of *att ta en fika*, or simply *fika* (coffee), into conference days.

Introducing *fika* breaks can provide residents with much-needed opportunities for relaxation and self-care. The Swedes are known for having a highly beneficial work and life balance compared to people in other countries.^4^ One proposed explanation is the culture of conscientiously taking regularly scheduled breaks, known as *fika*, during the workday to relax and regroup.^5^ That logic could be extrapolated into resident education. If residents could participate in *fika* and engage in pleasant conversations with time away from the intense learning environment, it could help alleviate stress, boost morale, and improve mental well-being.

In this study we explored the potential advantages of taking 15-minute *fika* breaks in conjunction with monitoring sleepiness levels using the Karolinska Sleepiness Scale (KSS). For the purposes of this study, four EM residency programs implemented *fika* breaks during resident conferences to assess whether taking a 15-minute *fika* break after the second hour of didactics impacted resident alertness. Our goal was to explore the concept of *fika* and how it may improve EM residents’ alertness during weekly conference.

## METHODS

### Study Design and Setting

We conducted a multicenter, randomized crossover trial from August 25, 2022–January 5, 2023 to determine the association between resident fatigue during conferences with and without a *fika* break among EM residents. Four EM residencies participated in this study, which was reviewed and approved by each hospital’s respective institutional review board. Table 1 outlines defining characteristics of the four sites.

**Table 1.** Participating residency program’s baseline characteristics.

Site number	Number of EM residents	Length of conference in hours	Scheduled breaks	Interactive sessions (small groups, flipped classes)
1	18	5	Yes	Yes
2	29	5	Yes	Yes
3	39	4–5	No^*^	Yes
4	24	4	Occasionally	Yes

* Food was available during conference.

EM, emergency medicine.

For fatigue assessment we used the KSS, a validated self-assessment tool used to measure an individual’s level of sleepiness or alertness at a given moment.^6^ Decreased levels of alertness using the KSS score have been associated with deceased performance and cognitive function.^7^^,^^8^ Developed by researchers at the Karolinska Institute in Sweden, the scale consists of a series of levels, typically ranging from 1–9, where each level corresponds to a different degree of sleepiness.^6^ Participants are asked to rate their current level of sleepiness based on the descriptions provided for each level. Lower numbers on the scale indicate higher levels of alertness (1 = extremely alert), while higher numbers indicate increasing levels of sleepiness (9 = very sleepy, great effort keeping awake, fighting sleep).^5^ The KSS is often used in sleep research, clinical settings, and studies related to fatigue and sleep disorders to gain insights into people’s subjective perception of their own alertness or drowsiness.^6^

Instructions were provided during intervention (*fika*) and control dates, asking the EM residents to circle the number that represented their perceived level of sleepiness at that point in time. An additional unrelated wellness question was included in the questionnaire to keep this study blind. During the first phase of the study, the four sites were split randomly into two groups. The two groups were then randomly assigned two control dates and two intervention dates. One group started with control dates, followed by intervention dates. The second group started with intervention dates, followed by control dates. This was done to help offset possible fatigue differences due to the passage of time. Table 2 demonstrates the control and intervention dates of each site.

**Table 2.** Dates of control and intervention by site.

Site number	Date of control	Date of intervention (Fika)
1	September 28, October 5	September 14, October 12
2	September 21, October 5	September 1, December 15
3	August 25, January 5	September 1, December 15
4	October 13, December 8	October 20, November 3

During the intervention days, a 15-minute Swedish-style *fika* break was added into the EM conference schedule after the second hour of conference. The fika breaks were to be held in a location outside the lecture area where EM residents were provided with coffee, non-caffeinated beverages, pastries, and snacks. Residents were instructed that there should not be any work-related discussion, as this break serves to encourage socialization and relaxing conversation. On control days, normal breaks occurred as scheduled during EM conference. During both phases, the survey was conducted before the last hour of conference.

### Selection of Participants

Study participants were EM residents across the four participating hospitals. There were 25 postgraduate (PGY) year-1 residents, 18 PGY-2 residents, 16 PGY-3 residents, and 4 PGY-4 residents for a total of 98 residents. A total of 64 residents participated in at least one survey during both the control and *fika* sessions to allow paired comparison.

### Interventions

The intervention in this study consisted of implementing a 15-minute *fika* break after the second hour of lecture where coffee, non-caffeinated beverages, pastries, and snacks were made available. This was conducted twice over several months. On control days, participating sites had instructions to not change any regular scheduled curriculum breaks or limit the food and drink that were normally present. Normal breaks at participating sites ranged from 10–15 minutes, and all programs had food and caffeinated drinks available on control days.

### Data Analysis

We compiled the numerical data obtained from the KSS surveys taken by the EM residents from the control and *fika* sessions for analysis and separated it into two subgroups, consisting of a residency site subgroup and a PGY subgroup. The residency site subgroup was broken down into each respective participating residency program, and the PGY subgroup was broken down into each PGY class (1–4). We used a paired sample *t*-test to compare the mean KSS of the resident cohort both before and after implementation of *fika*. This was done for individual EM residency programs and for all EM residency programs participating as a larger cohort.

## RESULTS

### Sleepiness on *Fika* vs Control Days

**Figure 1.**
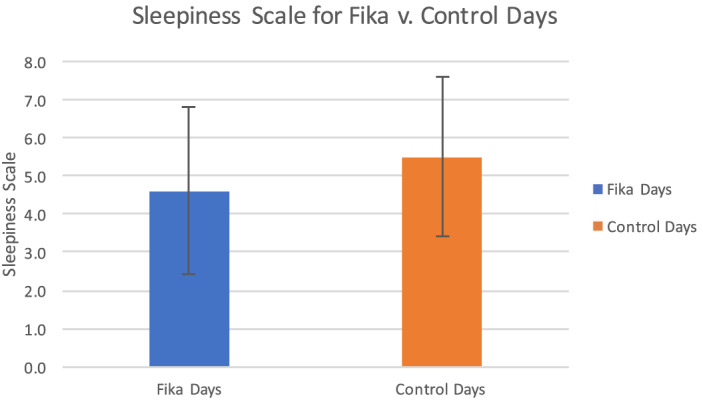
Mean Karolinska Sleepiness Scale scores for *Fika* vs control days. Mean sleepiness on days when *fika* was implemented was improved compared to control. Average sleepiness scale was 4.6 (SD 2.2) and 5.5 (SD 2.1), respectively, on *fika* and control days, respectively.

**Figure 2.**
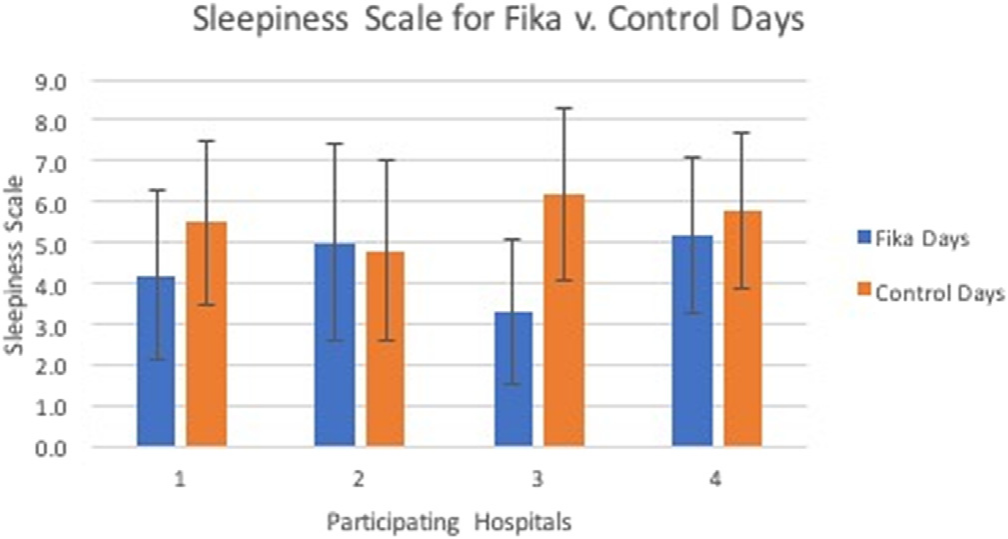
Results of self-reported Karolinska Sleepiness Scale scores on *fika* days vs control days, separated by participating residencies. Site 3 showed most improvement of sleepiness from the Fika intervention, with 3.3 (SD 2.1) on *fika* days vs 6.2 (SD 2.0) on control days, while Site 2 showed the opposite effect, with 5 (2.4) on *fika* days vs 4.8 (SD 2.2) on control days, although the difference was not statistically significant.

Figure 1 presents the mean results of the sleepiness measured on days where *fika* was implemented vs control. The average sleepiness was 4.6 on *fika* days and 5.5 on control days with standard deviation of 2.2 and 2.1, respectively, P-value = 0.004. This indicates that residents were more awake on days when *fika* was implemented, and this result was statistically significant. Figure 2 demonstrates the results of the KSS separated by participating residency programs. Site 3 had the biggest improvement in sleepiness (3.3 on *fika* days vs 6.2 on control days). Site 2 had the least improvement in alertness and actually showed that *fika* intervention increased sleepiness in residents (5.0 on *fika* days vs 4.8 on control days), although the difference was not statistically significant.

**Figure 3.**
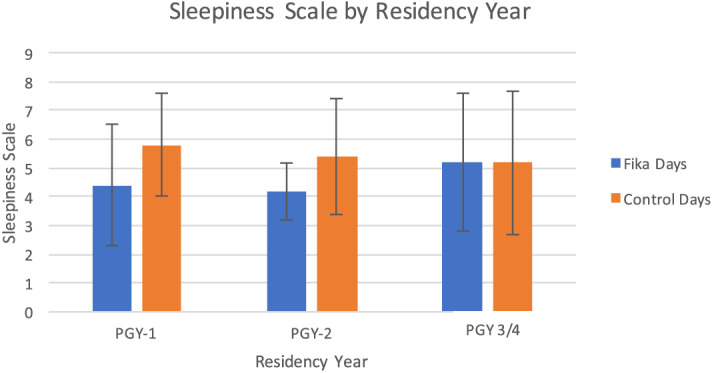
Sleepiness scale by residency year. Results show *fika* intervention had greatest improvement in sleepiness among PGY-1 residents, with sleepiness of 4.4 (SD 2.1) on *fika* days and 5.8 (SD 1.8) on control days, although this effect was not statistically significant due to small sample size.

Figure 3 depicts the alertness by residency year from all participating residency programs. We found that improvement in alertness was more visible in first- and second-year residents compared to third- and fourth-year residents. Due to low sample size, however, this difference was likely by chance and not statistically significant.

## DISCUSSION

The findings suggest that the inclusion of *fika* breaks into the EM residency didactics positively influenced participants’ sleepiness levels. The reduced sleepiness during conference sessions could potentially enhance residents’ attention, engagement, and knowledge retention, leading to improved educational outcomes.

### Improved Learning Outcomes

While the primary goal of residency conferences is to impart medical knowledge and skills, the effectiveness of learning can be enhanced by incorporating *fika* breaks and monitoring sleepiness levels with KSS. Studies have shown that brief breaks during learning sessions can improve attention and retention of information. By stepping away from conference lecture sessions and using the KSS, we were able to assess residents’ levels of sleepiness and determine the effectiveness of the breaks in reinvigorating their focus. These findings can be used to optimize the timing and duration of *fika* breaks, ensuring that they contribute to improved learning outcomes and better knowledge retention.

### Fostered Social Interactions

Building a strong sense of community and fostering social interactions is vital for resident overall well-being.^9^ *Fika* breaks provide an ideal platform for residents to connect on a more personal level, share experiences, and develop supportive relationships with their peers and faculty members. These informal interactions encourage open communication, collaboration, and mentorship opportunities. By also considering the sleepiness levels with KSS during these breaks, organizers can tailor the frequency and structure of *fika* sessions to promote optimal social interactions while mitigating the risk of residents becoming excessively fatigued or drowsy.

## LIMITATIONS

The study has several limitations, including a relatively small sample size and a short intervention period. Future research could involve larger studies with extended intervention periods to further explore the long-term effects of *fika* breaks on EM residency conference days. During control days, residents could have consumed coffee or soda that contained caffeine. Lasty, the effect of different type of breaks could also be considered, such as a walk or other intentional break, to determine whether that activity has the same effect. All participating sites on control days had slight variations in the nature and length of breaks.

## CONCLUSION

Incorporating Swedish-style *fika* breaks into emergency medicine residency conferences improved the overall alertness in EM residents. Residency programs should consider this unique approach to prioritize resident wellness and optimize educational experiences. Further research can explore the long-term effects of *fika* breaks and Karolinska Sleepiness Scale monitoring on resident burnout, performance, and career satisfaction in EM residency programs.
